# Trend and status of out-of-pocket payments for healthcare in Iran: equity and catastrophic effect

**DOI:** 10.1186/s42506-020-00055-w

**Published:** 2020-11-03

**Authors:** Satar Rezaei, Abraha Woldemichael, Mohammad Ebrahimi, Sina Ahmadi

**Affiliations:** 1 Research Center for Environmental Determinants of Health, Health Institute, Kermanshah University of Medical Sciences, Kermanshah, Iran; 2 Department of Health Systems, School of Public Health, College of Health Sciences, Mekelle University, Tigray, Ethiopia; 3 Student Research Committee, Kermanshah University of Medical Sciences, Kermanshah, Iran; 4 Department of Social Welfare Management, University of Social Welfare and Rehabilitation Sciences, Tehran, Iran

**Keywords:** Financing personal, Health equity, Health disparities, Out-of-pocket payments, Health expenditure, Kakwani progressivity index, Catastrophic healthcare expenditure

## Abstract

**Background:**

Equity in the distribution of health care resources and mitigating the risk of out-of-pocket (OOP) catastrophic healthcare expenditures (CHE) are the major objectives of the health system of a country. This study aims to measure equity in OOP payments for healthcare and the incidence of CHE among Iranian households over time.

**Methods:**

This retrospective cross-sectional study utilized data extracted from the household income and expenditure survey (HIES) of Iran, collected by the Statistical Center of Iran. The analysis included a total of 174,341 households’ five yearly data of 6 years starting from 1991 to 2017. Kakwani progressivity index (KPI) was used to measure the equity in OOP payment for each year and examine the households’ incidence of CHE at 20%, 30%, and 40% of their capacities to pay (CTP). The trend series regression analysis was used to examine the trend in the KPI and the incidence of the CHE over time.

**Results:**

The findings indicated that the households’ expenditure on health out of their monthly budgets for the years 1991 and 2017 were 2.1% and 10.1%, respectively. The KPI for the OOP payment was negative for all 6-year observations (1991 = − 0.680; 1996 = − 0.608; 2001 = − 0.554; 2006 = − 0.265; 2011 = − 0.225, and 2017 = − 0.207), indicating that the OOP payments for healthcare are regressive and more concentrated among the socioeconomically disadvantaged households. There was a statistically significant (*p* = 0.003) increase in the KPI (i.e., decline in the regressivity) over time. The incidence of the CHE (1.12, 1.93, and 3.71%) in 1991 at the CTP levels of 20%, 30%, and 40% was lower than the incidence at the corresponding levels of CTP (5.26, 10.88, and 22.16) in 2017. The findings of the time-series regression indicated a statistically significant (*p* < 0.05) increase in the incidence of the CHE at the 20%, 30%, and 40% levels of the households’ CTP.

**Conclusions:**

The current study demonstrated that OOP payment as a source of healthcare funding in Iran is inequitable. While the use of interventions such as the prepaid and publicly funded programs may contribute to the reduction of CHE and improvement of equity in healthcare financing, further inequality analyses in the incidence of the CHE among households and its main determinants can contribute to evidence-informed planning to reduce the CHE in the context.

## Introduction

The concern for equity in healthcare financing is a common issue in many countries regardless of their socioeconomic development [1–3]. A health system is equitable if all people have fair access to healthcare, and if the people’s ability to pay is not limiting their healthcare utilization. In healthcare, the perspective of equity study is either healthcare utilization or healthcare financing [4–6].

The sources to healthcare financing in many countries included general taxation, social health insurance, private health insurance, community financing, and out-of-pocket (OOP) payments [7]. In under-resourced countries like Iran, OOP healthcare financing is a major source of funding [8, 9]. In Iran, the OOP payment accounted for more than 50% of the total healthcare expenditure of the country [10] which is higher than the values reported for other countries [11]. The high OOP payment for healthcare is likely to be associated with CHE and inequity of healthcare financing [12]. A study indicated that more than 5% of the Iranian households experienced CHE, and the CHE was higher among those in lower socioeconomic status [13].

Households may suffer the consequences of OOP payments for two reasons. First, the direct OOP payment for the healthcare service at the time of service use can lead households to CHE. Second, the OOP payment for healthcare may be regressive. The unfair distribution of OOP payment for healthcare can negatively influence equity in healthcare financing, and this can be measured using the Kakwani progressivity index (KPI), which is one of the most commonly used methods [9, 14]. An increase in households’ OOP payment for healthcare services with an increase in income indicates a progressivity in OOP payment and an increase in OOP payment with a reduction in income shows a regressivity in OOP payment for healthcare [15]. In Iran, there is limited evidence on the trend of equity in healthcare financing. Few studies reported the existence of the inequity of healthcare financing. For example, one study reported a positive KPI and progressive OOP payments for healthcare for the urban residents and a negative KPI and regressive OOP for the rural ones [16]. Another study reported a negative (− 0.112) KPI for the healthcare costs (OOP payment for healthcare plus health insurance premiums) [17]. Besides, the KPI for general taxation and health insurance premiums were progressive and regressive, respectively [18].

Monitoring the equity of OOP payments for healthcare services and measuring the incidence of CHE among households over time are two main strategies to examine a health system’s performance towards financial protection for citizens. This study aims to measure the equity in OOP payments for healthcare services and determine the incidence of CHE at the households’ 20%, 30%, and 40% levels of CTP using five yearly national household survey data for 6 years. The findings of this study are anticipated to contribute valuable input for policymakers in Iran and other countries with similar contexts to ensure equity in healthcare financing and reducing CHE.

## Methods

### Study setting

Iran is a developing country, located in the Eastern Mediterranean Region (EMR) with 80 million population living across 31 provinces in urban (76%) and rural (24%) settings https://www.amar.org.ir/english/Population-and-Housing-Censuses.

### Study population, sampling method, data collection, and sample size

This retrospective cross-sectional study was conducted using data extracted from the household income and expenditure surveys (HIESs) of Iran, which was collected annually by the Iranian Statistical Center (ISC) https://www.amar.org.ir/english/Statistics-by-Topic/Household-Expenditure-and-Income#2220530-releases. In summary, in the HIESs, the data was collected using a structured standard questionnaire for a face-to-face interview for the household head. All data in the HIES were collected for the past month before the survey from the households included in the survey. All the households living in the rural and urban areas of Iran were eligible to participate in the survey. A multistage cluster sampling technique was applied to obtain the sample. Finally, after excluding the observation with incomplete information, a total of 174,341 households for 6-year observations for the years 1991 (*n* = 18 582), 1996 (*n* = 21 854), 2001 (*n* = 26 714), 2006 (*n* = 31 111), 2011 (*n* = 38 220), and 2017 (*n* = 37 860) were included in the study.

### Data and variables

The household size and total monthly expenditure in the last month before the survey were the variables used for the analysis. We used the household expenditure on health as the total OOP payment for healthcare services, and the total expenditure (TE) as a proxy for the households’ ability to pay [6, 19, 20]. The household expenditure is more advantageous than the household income because income is likely to be underreported and can vary over time [19, 20]. As per previous studies and for the proper comparison [6, 21], we equivalized the households’ TE and OOP payment by dividing the TE and OOP payment to the squared root of the household size.

### Measuring progressivity in OOP payment for healthcare

Like other previous studies [6, 22], we measured the KPI that was used to measure the equity in OOP payments for healthcare among Iranian households using five yearly data points of 6 years (1991, 1996, 2001, 2006, 2011, and 2017), and mathematically, this is expressed as follows [22, 23]:

$$ \mathrm{KPI}={C}_{\mathrm{oop}}-{G}_{\mathrm{atp}} $$

where KPI represents the Kakwani progressivity index, *C* is the concentration index for the OOP payment for healthcare, and *G* represents the Gini coefficient for the household’s income.

The values of *C* can range from − 1 to +1. The negative (positive) value indicates the concentration of the OOP payment in favor of the socioeconomically disadvantaged (advantaged) households, while the zero value indicates perfect equality. The *G* values range from 0 to 1, and the farther away the value from zero, the higher is the inequality. Generally, the *C* (*G*) is twice the area between the concentration (Lorenz) curve and the line of perfect equality. The KPI values range from − 2 to 1. The negative KPI values for the OOP payment show the regressivity of the OOP payment for healthcare, indicating an inverse relationship between the OOP payment for healthcare and the household’s income. That is, an increase in the households’ income will be associated with a decrease in the OOP and *vice versa*. A positive KPI indicates the progressivity of the OOP payment for healthcare, suggesting the existence of equity in the OOP payments, and an association between the OOP payment for healthcare and households’ income. If OOP payment is proportional to household’s income, the KPI is zero [6, 16].

### Measuring catastrophic healthcare expenditure

This study applied the World Health Organization’s (WHO) standard approach to calculating the incidence of CHE at 20%, 30%, and 40% of the households’ capacity to pay (CTP) https://www.amar.org.ir/english/Population-and-Housing-Censuses. In this study, we described the CTP of the household as the difference between the households’ total expenditure (TE) and the expenditures of food adjusted to household size [13, 19]. For example, at the 40% of the households’ CTP, a household is considered in a state of CHE if the household’s OOP payment is greater than or equal to 40% of its CTP. The more details of the calculation of CHE base on WHO approach are available elsewhere [24, 25].

### Trend analysis

We conducted a 6-year time-series observation regression analysis for the KPIs and incidence of the CHE and presented the findings for the total, urban, and rural households. In addition, we performed a trend regression analysis of the incidence of CHE at 20%, 30%, and 40% levels of the household’s CTP separately. When the coefficient value is positive and its *p* value is less than 0.05, it indicates an increasing and statistically significant trend in progressivity (incidence of CHE) over time and a negative value indicates a statistically significant decreasing trend.

## Results

### Ability to pay and out-of-pocket payment for health care

The mean equivalized households’ TE and OOP for healthcare for the total, urban, and rural households in Iran for each year is summarized in Table 1. Overall, the mean equivalized TE of the households increased from 182,655 Iranian Rials (IRR) in 19991 to 10,313,116 IRR in 2017. Besides, the trend in households’ TE of the total, urban, and rural households showed a statistically significant (*p* < 0.05) increase overtime. However, the mean equivalized TE for urban households was higher than the rural ones across the years. Despite the increasing trend in the mean equivalized OOP payment for healthcare for the total, urban, and rural households over time, the increasing trend was significant (*p* = 0.036) for the total households.

**Table 1:** Mean equivalized TE and OOP payment for health care of the total, urban, and rural households over 6 years in Iran

Survey year	Mean E.TE total (SD)	Mean E.TE urban (SD)	Mean E.TE rural (SD)
1991	182,655 (473,718)	137,687 (588,989)	226,166 (306,352)
1996	362,793 (370,750)	469,789 (447,086)	255,600 (227,952)
2001	997,484 (1,469,453)	1,332,746 (1,967,313)	710,833 (719,879)
2006	2,453,703 (3,042,291)	3,161,533 (326,331)	1,843,366 (3,793,022)
2011	5,300,166 (5,115,093)	6,186,995 (4,116,561)	4,464,271 (5,866,648)
2017	10,313,116 (8,955,380)	12,927,756 (6,392,161)	7,773,356 (1,040,000)
Trend coefficients (*p* value)	1,912,018 (0.012)	2,285,321 (0.040)	1,471,271 (0.011)
**Survey year**	**Mean E.OOP payment for healthcare total (SD)**	**Mean E.OOP payment for healthcare urban (SD)**	**Mean E.OOP payment for healthcare rural (SD)**
1991	3825 (24,939)	4632 (29,528)	3044 (19,465)
1996	17,673 (93,656)	22,382 (110,431)	12,956 (72,786)
2001	59,020 (548,010)	80,580 (791,694)	40,586 (143,075)
2006	165,244 (426,211)	217,035 (2,006,705)	120,585 (492,108)
2011	362,151 (675,707)	436,793 (789,606)	291,796 (537,671)
2017	1,094,500 (2,070,867)	1,373,426 (2,374,534)	823,561 (1,681,805)
Trend coefficients (*p* value)	188,372.3 (0.036)	319,090 (0.337)	143,403 (0.330)

*E* equivalized, *SD* standard deviation, *TE* total expenditure, *OOP* out-of-pocket

**Fig. 1:**
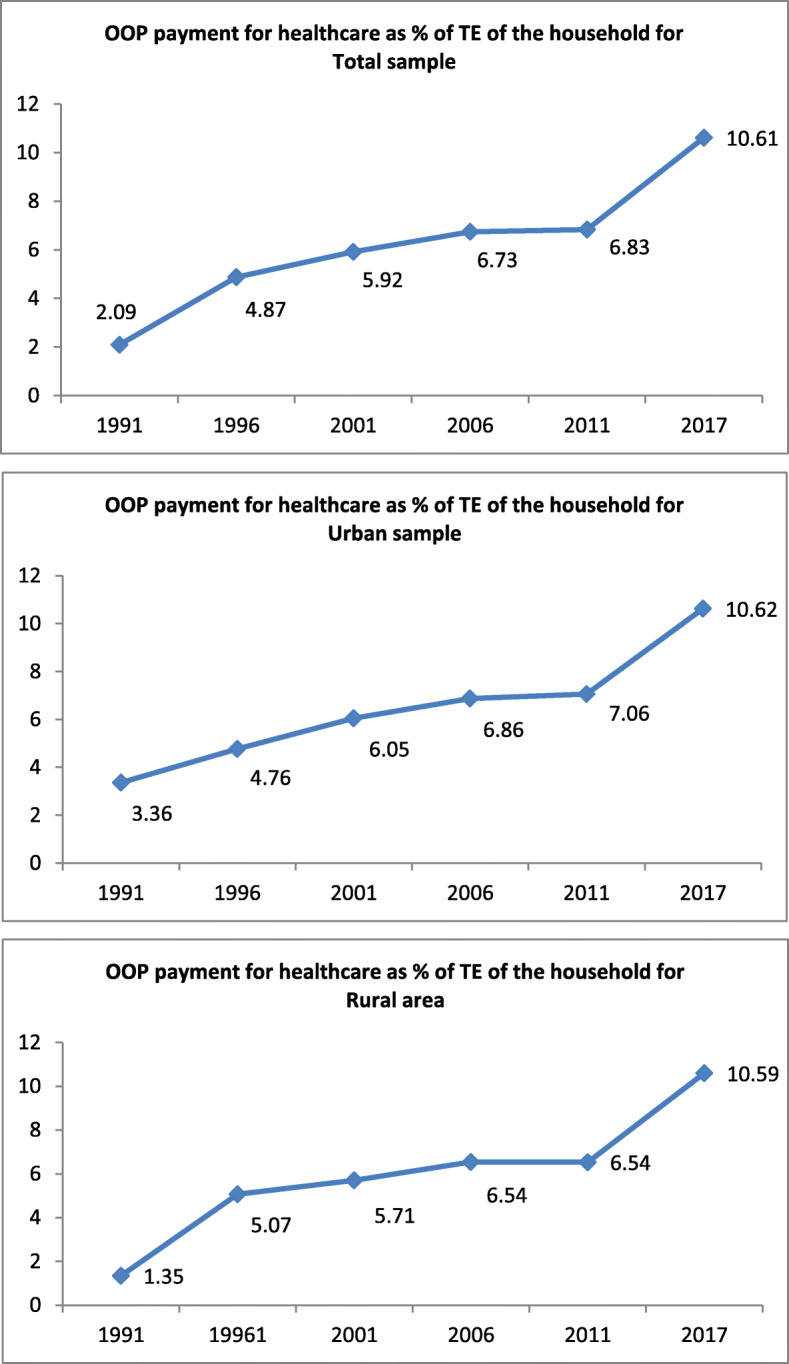
Trend in OOP payment for healthcare services as percentage of the households’ CTP for the total, urban, and rural households over six years in Iran. Note: TE refers total expenditure

The percentage of households’ OOP payments for healthcare out of the TE for total, rural, and urban households is indicated in Fig. 1. The OOP payment for total households in 1991 accounted for 2.09% of the TE, while in 2017 it accounted for 10.61%. Despite a considerable difference between the urban and rural households (3.36% versus 1.35%) in the percentage of OPP payment for healthcare during 1991, there was no marked difference between the two (10.62 versus 10.59%) in 2017.

### Progressivity in out-of-pocket health expenditure

The KPIs of the households’ OOP payments for the total, urban, and rural households across the years were negative (Table 2), suggesting the regressivity of the OOP payment. That is, the households’ OOP payment for healthcare relative to their CTP was not equitable, and there was an inverse relationship between the OOP payment for healthcare services and the TE of the households. The KPI for total households increased from − 0.680 in 1991 to − 0.207 in 2017. The regression analysis of the KPIs time-series observations for the total, urban, and rural households showed statistically significant (*p* < 0.05) declining trends in the regressivity of the households’ OOP payments, and the three coefficients were nearly equal.

**Table 2:** KPIs for total, urban, and rural households over 6 years in Iran

Survey year	KPIs for total households	KPIs for urban	KPIs for rural
1991	− 0.680	− 0.560	− 0.741
1996	− 0.608	− 0.600	− 0.544
2001	− 0.554	− 0.586	− 0.442
2006	− 0.265	− 0.199	− 0.319
2011	− 0.225	− 0.219	− 0.209
2017	− 0.207	− 0.189	− 0.165
Trend coefficients (*p* value)	0.1086 (0.003)	0.967 (0.025)	0.1144 (0.001)

*KPIs* Kakwani progressivity index

### Catastrophic healthcare expenditure

The incidence of the CHE for total, urban, and rural households at 20%, 30%, and 40% levels of their CTP is presented in Table 3. At the 40% level of threshold of household CTP, the incidence of CHE for the total, urban, and rural households in 1991 were 1.12, 1.15, and 1.09%, respectively. The figures for the corresponding categories in 2017 were 5.26, 4.02, and 6.47% in 2017, respectively. The time-series regression of incidence of CHE for total, urban, and rural households at the 40% level of the CTP revealed a statistically insignificant (*p* > 0.05) increasing trend in the incidence. At the 30% level CTP, the trend for the CHE was statistically insignificant (*p* = 0.136) only for urban households, while at the 20% level, the trends for all three categories increased significantly (*p* < 0.05) over time.

**Table 3:** Incidence of CHE at the three different levels of the CTP of the households for total, urban, and rural areas over 6 years in Iran

Survey year		CHE for total	CHE for urban	CHE for rural
1991	40% thresholds	1.12	1.15	1.09
	30% thresholds	1.93	1.98	1.88
	20% thresholds	3.71	3.91	3.53
1996	40% thresholds	2.42	1.93	2.91
	30% thresholds	3.98	3.18	4.77
	20% thresholds	7.38	5.69	9.07
2001	40% thresholds	4.08	4.09	4.07
	30% thresholds	6.75	6.54	6.96
	20% thresholds	13.04	12.40	13.67
2006	40% thresholds	1.75	1.33	2.11
	30% thresholds	3.72	1.08	6.00
	20% thresholds	10.48	9.17	11.61
2011	40% thresholds	3.38	3.76	3.03
	30% thresholds	6.43	6.85	6.04
	20% thresholds	13.86	14.77	13.01
2017	40% thresholds	5.26	4.02	6.47
	30% thresholds	10.88	8.63	13.07
	20% thresholds	22.16	19.49	24.76
Trend coefficients (*p* value)	40% thresholds	0.607 (0.094)	0.488 (0.157)	0.723 (0.100)
	30% thresholds	1.402 (0.039)	1.10 (0.136)	1.06 (0.032)
	20% thresholds	3.118 (0.009)	2.91 (0.006)	3.31 (0.019)

*CTP* capacity of pay, *CHE* catastrophic healthcare expenditure

## Discussion

The current study indicated that the households’ expenditure on health out of their monthly budgets for the years 1991 and 2017 increased 5 times in Iran. The KPI for the OOP payment was negative for all 6-year observations, indicating that the OOP payments for healthcare are regressive and more concentrated among the socioeconomically disadvantaged households. There was a statistically significant increase in the KPI (i.e., decline in the regressivity) over time. The incidence of the CHE in 1991 at the CTP levels of 20%, 30%, and 40% was lower than the incidence at the corresponding levels of CTP in 2017. The findings of the time-series regression indicated a statistically significant increase in the incidence of the CHE at the different levels of the households’ CTP.

The use of income as a proxy for the households’ ability to pay is not appropriate because income varies from time to time and tends to overestimate the households’ expenditures [6, 19, 20]. Our study applied the total expenditure in determining the ability to pay of the household. The findings indicated an increasing trend in the OOP payment for healthcare over time. Another study in Western Iran also reported a higher expenditure (5% versus 9%) in 1991 and in 2011 [26].

Our findings also indicated negative coefficients of the KPIs for the OOP payment for healthcare across the years and the categories, indicating the regressivity of the households’ OOP payments for healthcare services. These findings are consistent with the reports of other several studies [6, 16–18, 27]. Another study in Africa also reported a negative coefficient (KPI = − 0.20) of the OOP for healthcare [28]. The regressivity of OOP payment for healthcare indicates that the OOP payment is not related to the households’ income. Similarly, another study in Ghana indicated regressivity for the OOP payment for healthcare and progressivity for taxation [29]. The different reforms in the health sector of Iran as the urban inpatient health insurance plan, the rural health insurance scheme, and the health sector evolution plan, which were introduced in 2001, 2005, and 2014, respectively might have contributed to the reduction in regressivity of the households’ OOP payments for healthcare services observed in our findings.

The incidence of CHE observed in our findings not only was high but also showed an increasing trend over time. Our findings indicated higher overall CHE than the findings of another study report [13]. The high CHE observed in our findings regardless of the residence of the households affects fair utilization of healthcare services among citizens, especially the disadvantaged groups, and threatens the achievement of sustainable health development goals for health in Iran. This situation calls for an effective health policy and an intersectoral effort to improve the socioeconomic status of the citizens to protect them against CHE and its negative consequences.

The difference observed in the incidence of the CHE among the urban and rural households observed in our study are almost consistent with the findings of several studies [2, 12, 30, 31]. The higher incidence of CHE among rural residents may imply poorer socioeconomic status of the rural households than the urban ones. Other researchers also reported that the OOP payments for healthcare led the poor rural people to be poorer than urban residents [30]. Unlike the findings in our study, independent studies in Portugal [32] and Canada [6] revealed a reducing trend in the incidence of CHE over time.

### Strengths and limitations of the study

Unlike previous studies in Iran based on 1-year data [18] or for a single province [17], our study analyzed the equity in OOP payments for healthcare services and its catastrophic incidence using 6-year annual data points representing the whole Iran and also using WHO standard approach. The overall picture of the total, urban, and rural households’ OOP payments and the incidence of the CHE at different levels of the CTP over time shed light to understand the effectiveness of existing policies in the context. Thus, the findings provided an important input for policy makers to make appropriate reforms to increase citizens’ financial protection against CHE in Iran. However, this study has some limitations. The household income and expenditure survey (HIES) data are self-reported and represent only the households’ OOP payment for healthcare of the past 1 month of the date of data collection, and it is prone to recall bias. Besides, this study did not analyze the equity status among households in relation to other sources of healthcare financing such as the general taxation and health insurance premiums because of the lack of data concerning these financing sources.

## Conclusions

Measuring the extent and trends of households’ OOP payment for healthcare services and CHE helps tracking the effect of existing health policies in reducing inequity in healthcare access to people. This study analyzed the progressivity/regressivity of OOP payments for healthcare services among Iranian households in relation to their income. The findings indicated increasing trends and considerably high incidence of CHE over time, implying the need for policymakers to pay emphasis to the citizens’ financial protection against CHE in order to ensure progress toward achieving the universal health coverage target of the sustainable development goals for health. While the use of policies such as the prepaid and publicly funded schemes may contribute to the reduction of high CHE and in improving equity in healthcare financing, further inequality analyses in the incidence of the CHE among households and its main determinants can contribute to evidence-informed planning to reduce the CHE in the context.

## Data Availability

The data used in the study was extracted from the household income and expenditure surveys (HIESs) collected by the Iranian Statistical Center (ISC). The HIES are publicly available at https://www.amar.org.ir/english/Statistics-by-Topic/Household-Expenditure-and-Income#2220530-releases.

## Abbreviations

CHE: Catastrophic healthcare expenditure
TE: Total expenditure
CTP: Capacity to pay
KPI: Kakwani progressivity index
HIES: Households’ income and expenditure survey
